# The role of community healthcare professionals in discussing sexual assault experiences during obstetrics and gynecological healthcare appointments

**DOI:** 10.1186/s12905-023-02401-4

**Published:** 2023-05-15

**Authors:** Andrea L. DeMaria, Stephanie Meier, Hannah King, Haley Sidorowicz, Kathryn C. Seigfried-Spellar, Laura M. Schwab-Reese

**Affiliations:** 1 Department of Public Health, College of Health and Human Sciences, Purdue University, 812 West State Street, 47907 West Lafayette, IN USA; 2 Division of Consumer Science, College of Health and Human Sciences, Purdue University, West Lafayette, IN USA; 3 Department of Biological Sciences, College of Science, Purdue University, West Lafayette, IN USA; 4 Department of Computer and Information Technology, Polytechnic Institute, Purdue University, West Lafayette, IN USA

**Keywords:** Sexual assault, Reproductive health, Qualitative methodologies, USA

## Abstract

**Background:**

Nearly half of adult women in the US report experiencing sexual assault, with almost one-fifth reporting rape. For many sexual assault survivors, healthcare professionals are the first point of contact and disclosure. This study aimed to understand how healthcare professionals working in community settings perceived their role in discussing sexual violence experiences with women during obstetrical and gynecological healthcare appointments. The secondary purpose was to compare healthcare professionals’ perspectives with the patients’ to determine how sexual violence conversations should occur in these environments.

**Methods:**

Data were collected in two phases. Phase 1 consisted of 6 focus groups (Sept-Dec, 2019) with women aged 18–45 (n = 22) living in Indiana who sought community-based or private healthcare for women’s reproductive healthcare needs. Phase 2 included 20 key-informant interviews with non-physician healthcare professionals (i.e., NP, RN, CNM, doula, pharmacist, chiropractor) living in Indiana (September 2019-May 2020) who provided community-based women’s reproductive healthcare. Focus groups and interviews were audio-recorded, transcribed, and analyzed using thematic analyses. HyperRESEARCH assisted in data management and organization.

**Results:**

There were three resulting themes: (1) healthcare professionals’ approaches to screening for a history of sexual violence varied depending on how they ask, what setting they work in, and type of professional asking; (2) healthcare experiences can compound traumatic experiences and create distrust with survivors; and (3) sexual violence impacts patient healthcare experiences through what services they seek, how professionals may interact with them, and what professionals they are willing to utilize.

**Conclusions:**

Findings offered insight into actionable and practical strategies for enhancing sexual violence screening and discussions in community-based women’s reproductive health settings. The findings offer strategies to address barriers and facilitators among community healthcare professionals and the people they serve. Incorporating healthcare professional and patient experiences and preferences for violence-related discussions during obstetrical and gynecological healthcare appointments can assist in violence prevention efforts, improve patient-professional rapport, and yield better health outcomes.

## Introduction

Nearly half of adult women in the US report experiencing sexual assault, with almost one-fifth reporting rape [1, 2], and many occuring postpartum [3]. Sexual abuse, in particular, is a risk factor for myriad conditions, including chronic and autoimmune diseases (e.g., irritable bowel syndrome, fibromyalgia, cardiovascular disease, chronic pelvic pain; [4, 5], mental health conditions (e.g., anxiety, depression, post-traumatic stress disorder, eating disorders; [6–14], and risky health behaviors (e.g., increased alcohol and tobacco use) [8]. Sexual assault negatively impacts reproductive and sexual health, with survivors being less likely to access and maintain routine gynecological assistance, including contraception care[3, 15], and having an increased risk for repeat unintended pregnancy and abortion [16–23]. Additionally, sexual assault survivors are more likely to experience recurring sexually transmitted infections.[15, 19, 24].

For many sexual assault survivors, healthcare professionals are the first point of contact and disclosure [20, 25]. These patients may visit healthcare offices more frequently due to increased health-related needs [14, 26], spending more money on healthcare services [10, 27]. The obstetrician-gynecologist medical community [28], including the American College of Obstetricians and Gynecologists (ACOG), recommends routine screening for sexual and interpersonal violence during obstetrical and gynecological healthcare appointments [29] and have created a set of guidelines for trauma-informed care [30]. However, research suggests healthcare professionals rarely screen for sexual abuse or discuss sexual abuse history [11, 14, 31, 32]. In one recent study, only 2% of women were asked about a sexual assault history by professionals in healthcare settings [33], which supported additional research noting limited sexual assault-related discussions with unprepared healthcare providers during routine visits [20, 26, 28, 34–42]. Yet, patients supported being screened [43–45], which helped them build trust with professionals and comfort with exam procedures [46, 47].

Despite patients’ support for screening, healthcare professionals may not be adequately trained to screen for or have conversations about sexual assault experiences [40, 48]. As a result, they often respond to disclosures rather than initiate discussions [49–51]. Making sexual assault screening a routine part of obstetrical and gynecological healthcare appointments may reduce distress or discomfort during examinations [40, 52]. Routine screening among pregnant and non-pregnant patients and subsequent intervention have demonstrated positive health outcomes, including early identification of unmet needs for timely referrals [53], decreased depression, improved pregnancy outcomes [54], and greater follow-up support [55]. However, healthcare professionals may need more education to emphasize the importance of screening [56], make appropriate referrals to community-based resources [56, 57], and handle the dynamic responses from patients who have experienced sexual assault. The absence of protocols and policies surrounding sexual assault screening presents a deficiency within obstetrical and gynecological and primary care settings, and it is important to understand how healthcare professionals navigate these insufficiencies. Further, there is a need for trauma-informed care approaches within obstetrical and gynecological healthcare [46, 50, 51, 58, 59] that go beyond the immediate care after an act of violence and extend to long-term medical consequences, especially those impacting sexual and reproductive health [58, 60].

In the gynecological setting, trauma-informed care, which requires professionals to understand the broad impacts of trauma and treat patients accordingly [61], could include changes to how they capture patient history, solicit consent before conducting physical exams, maintain confidentiality of information, and connect patients to wraparound and interdisciplinary team-based services [8, 60, 62]. Trauma-informed care has two components: universal trauma precautions and trauma-specific care [51]. Universal trauma precautions are implemented in all settings regardless of patient trauma history [58]. Utilizing this strategy and its precautionary measures may help build patient trust [51]. Establishing trust is critical because sexual assault survivors are more likely to share their experiences if they have a positive relationship with their healthcare professional [63]. The second component, trauma-specific care, occurs when professionals are aware of a history of trauma and modify their approach to care, such as providing interprofessional care, delivering more targeted services, and using a holistic approach [51]. Trauma-specific care may also emphasize empowerment, social connectedness, and self-esteem [64, 65].

Patient-centered, trauma-informed care during obstetrical and gynecological healthcare appointments may create outlets for disclosure and improve overall reproductive health and well-being [20]. Screening may be particularly important during the COVID-19 pandemic as the social distancing requirements have increased women’s risk for violence- and mental-health-related concerns [66]. Due to the wide range of strategies and the individuality of screening for sexual assault, it is necessary to understand how healthcare professionals interact with their patients in the context of sexual assault assessments and care, along with their perspectives on their approaches.

### Study purpose

This study aimed to understand how healthcare professionals working in community settings perceived their role in discussing sexual assault during obstetrical and gynecological healthcare appointments. A secondary purpose was to compare healthcare professionals’ perspectives with those of the patient to determine how sexual assault conversations are occurring within these settings.

## Method

Focus group discussions with reproductive-aged women and in-depth interviews with non-physician healthcare professionals practicing in community settings were conducted as part of a larger study on reproductive healthcare in community settings [67]. The discussions offered insight into group dynamics, which can be particularly impactful when exploring shared experiences among participants [68]. Because women’s reproductive health decisions may depend on lifestyle and context, focus groups provided an effective way to understand how women constructed sexual assault in the broader context of their lives [68]. The interviews offered insight into how sexual assault-related discussions occur within obstetrical and gynecological healthcare appointments. The first author’s university approved all protocols and procedures for this study (IRB-2019-160).

### Focus group discussions with reproductive-aged women

We completed six semi-structured focus groups with 2–9 participants each (*n* = 22), ranging 88–131 min, in September-December 2019. Women were eligible to participate in the study if they were 18–45 years of age and had ever sought reproductive healthcare at a community health center or program in Indiana. We recruited through flyers at community health centers and in public locations (e.g., community centers, libraries), emails through community health service listservs, shareable social media advertisements, and in-person recruitment with onsite sign-up sheets. Community partners assisted recruitment efforts in three Indiana counties (Tippecanoe, Montgomery, Marion), which were selected based on demographic and geographic makeup (i.e., race/ethnicity, rurality), established connections, and convenience. Theoretical and snowball sampling, which included referrals from participants for other eligible women, improved data robustness and increased community member inclusion [69]. Following each focus group, participants completed an anonymous demographic survey.

Focus groups were conducted in locations convenient to the participants and researchers (e.g., reserved, private conference room in a community building). All participants received a $25 gift card to compensate their time, and all participants provided informed consent for participation and audio-recording. Each focus group was led by a moderator (the second author) and a co-moderator, who were both trained in graduate-level qualitative methodology. All discussions were conducted using a semi-structured protocol, allowing participant experiences and social dynamics to drive the conversation and researchers to explore unique perspectives, novel experiences, and shared knowledge [68, 69]. Focus groups began with general questions about participants’ health to build rapport and facilitate inter-participant discussions [69]. Researchers then discussed reproductive health experiences and sexual assault perspectives (Table 1). Focus groups continued until data reached theoretical saturation (i.e., when study categories and themes were stabilized and reinforced rather than further explained with incoming data; [70]. Participant characteristics are noted in Table 2.

**Table 1 This table shows up weirdly formatted in the PDF version. Can you double check it and fix the spacing of the questions in the left column?:** Focus group and interview questions

FOGUS GROUP QUESTIONS	FOCUS GROUP PROBES
How might sexual violence affect how women make decisions about their body?	For example, about birth control or pregnancy?
	Do you think this would affect women? Why or why not?
	What about coercive behaviors by a partner, such as deliberately breaking a condom or being convinced to do something they didn’t want to do?
	Who do you think has control over women’s choices about their health, specifically their reproductive and sexual health? Why?
	Have you or someone you know ever had an experience with sexual abuse or violence that affected decisions you made about your reproductive or sexual health choices (e.g., birth control, pregnancy, etc.) How?
Has a healthcare provider ever asked you or someone you know about sexual violence or sexual abuse experiences when talking about sexual or reproductive health?	Do you think healthcare providers should ask about this? Why or why not?
	Do you think this would be a good thing, a bad thing? Why or why not?
	If yes, how did this make you feel?
	If not, do you wish they would have? Why or why not?
**INTERVIEW QUESTIONS**	**INTERVIEW PROBES**
What do you perceive is the provider’s role in discussing sexual violence experience with women in community reproductive healthcare?	Why do you think this? Do you think these experiences play a role in women’s reproductive health decision-making? Why or why not? Do you think these experiences are important in community health settings, specifically? Why or why not?
Have you ever discussed a patient’s sexual violence experience during a reproductive health consultation? Why or why not?	[if yes] Please describe what leads to these conversations? [if yes] How do you broach the subject of sexual violence with women? [if yes] How do these conversations go? Why do you think so? [if no] Can you share why you haven’t had these conversations?

**Table 2:** Focus group participant characteristics

	n = 22
**Age**	25.8 ± 5.3
**Race Ethnicity**	
White/Caucasian	12 (54.5%)
Black/African American	7 (31.8%)
**Education**	
Some College or Undergraduate Degree	13 (59.1%)
Graduate Degree	5 (22.7%)
**Employment**	
Student	10 (45.5%)
Full-Time	11 (50.0%)
**Insurance**	
Private	17 (77.3%)
Public	3 (13.6%)
**Relationship Status**	
Single	19 (86.4%)
**Primary Birth Control**	
None	8 (36.3%)
Pill	4 (18.2%)
IUD	4 (18.2%)
Condoms	4 (18.2%)
**Had Been Pregnant**	6 (27.3%)

*Listed as n(%) or Mean ± Standard Deviation. Items that do not add up to 100% reflect missing data*

### In-depth interviews with healthcare professionals

We conducted 20 in-depth interviews with non-physician healthcare professionals (i.e., registered nurses (RN), nurse practitioners (NP), certified nurse-midwives (CNM), clinical pharmacists, doulas, and a chiropractor) who provided reproductive healthcare in Indiana (September 2019-March 2020). Inclusion criteria were: (1) working in a community healthcare setting, and (2) being a non-physician healthcare professional. The chiropractor was included because she served in community health settings and treated pregnant and postpartum women in concert with CNMs and doulas. Interviewees were recruited via purposive and snowball sampling, including email invitations to healthcare professionals from community partners (i.e., clinic office managers, agency directors, public health practitioners). Interviews continued until data saturation was reached for each thematic category [71, 72].

Interviews occurred at the participant’s convenience, either in person or via phone. All participants provided informed consent for participation and audio-recording. Interviews were audio-recorded and lasted between 49 and 91 min. Interviews were conducted by the second author and followed a semi-structured approach allowing interviewees to present novel concepts and discuss their experiences and knowledge holistically [69, 70]. Interviews began with rapport-building questions to enhance comfort and sharing before inquiring into community-based reproductive healthcare provision experiences. Following a discussion of patient-care experiences, the interviews transitioned to sexual assault, including perceived role of healthcare professionals in having sexual assault-related conversations (Table 1). All participants completed a brief demographic survey (Table 3) at the end of the interview, and they did not receive an incentive for participating.

**Table 3:** Interview participant characteristics

	n = 20
**Gender**	
Woman	19 (95.0%)
Man	1 (5.0%)
**Age**	39.7 ± 12.7
**Years in Women’s Reproductive Healthcare**	9.5 ± 8.7
**Years Serving Current Community**	11.2 ± 10.3
**Healthcare Organization Type**	
Community-Based Program	10 (50.0%)
Health System	3 (15.0%)
University Affiliated	2 (10.0%)
Private Organization/Agency	5 (25.0%)
**Professional Title**	
Nurse Practitioner	7 (35.0%)
Registered Nurse	3 (15.0%)
Certified Nurse Midwife	2 (10.0%)
Nurse Practitioner/Certified Nurse Midwife	1 (5.0%)
Clinical Pharmacist	3 (15.0%)
Doula	3 (15.0%)
Pregnancy & Postpartum Chiropractor	1 (5.0%)
**Specialty**	
Primary/Family Practice	7 (35.0%)
Obstetrics/Gynecology	13 (65.0%)
**Race Ethnicity**	
White/Caucasian	18 (90.0%)
Latino/a	1 (5.0%)
Black/African American	1 (5.0%)
**Education**	
Associate’s Degree/Some College	3 (15.0%)
Undergraduate Degree	1 (5.0%)
Graduate Degree	16 (80.0%)
**Location**	
Urban	12 (60.0%)
Rural	8 (40.0%)

*Listed as n(%) or Mean ± Standard Deviation*

### Data analysis

We utilized thematic analysis, a widely used, accessible, and flexible framework for analyzing qualitative data through identification, organization, and analysis in systematic phases [73, 74]. First, we conducted immersive, full content review to ensure familiarity with all data [73]. During this phase, we noted immediate patterns or ideas for potential codes and themes [73]. Following familiarization, we utilized a deductive/inductive approach for codebook development to allow greater data representation during the coding process [75, 76]. Initial codes were generated deductively and compiled into a preliminary codebook draft [76]. The inductive component permitted us to modify or add codes to better capture emerging themes from participant responses [76]. Coding was performed using HyperRESEARCH 4.5.1 [77]. Multiple rounds of coding were conducted until saturation was reached (i.e., no additional new codes were added to the data set) [73]. The first, third, and fourth authors collated data into potential themes and subthemes. Theme development was data-driven and closely reflected participant responses [78]. All co-authors reviewed resulting candidate themes. We thoroughly and collaboratively discussed and analyzed individual themes and incorporated relevant subthemes to provide structure and differentiate levels of meaning [73]. Any discrepancies were resolved via consensus discussion and data review until the final themes were fully agreed upon.

## Results

There were three resulting themes: (1) healthcare professionals’ approaches to screening for a history of sexual assault varied depending on the providers’ work setting and their field; (2) healthcare experiences can compound traumatic experiences and create professional distrust with survivors; and (3) sexual assault impacts patient healthcare experiences through the services they seek, how professionals interact with them, and type of professional they are willing to see. Quotes are presented with focus group number (FGX) or healthcare professional specialty (e.g., NP, doula, CNM).

### Screening for sexual assault: “I mean it’s mainly domestic violence screening… not really sexual assault screening.” (NP)

Healthcare professionals described their approach to screening patients about sexual assault. Focus group participants discussed the impact their healthcare professional’s approach had on their healthcare experience.

***Screening experiences and perspectives***. Interviewees had a variety of responses when asked if they screen for a history of sexual assault. These professionals’ responses signify the variation in sexual assault screening. Other forms of screening, such as probing questions during in-person exams, were noted. Interviewees utilized questionnaires to guide them during the screening process and to prudently navigate sexual assault screening. Their careful navigation of sexual assault screening, and continued efforts while with a patient, illustrate the caution and persistence necessary by professionals when performing sexual assault assessments. Focus group discussions also emphasized an absence of screening for sexual assault. Overall, narratives emphasized the lack of sexual assault screening taking place in obstetrical and gynecological appointments while also highlighting healthcare professionals’ experiences during assessments.

“For a very first visit, I probably wouldn’t even though, you could definitely make an argument that you should.” (NP)

“I mean it’s mainly domestic violence screening… not really sexual assault screening. I don’t think… I doubt very many providers ask [about sexual assault].” (NP)

“Typically if I just have a patient in front of me…we have like a little questionnaire thing that we kind of go through.” (NP)

“That’s something that we ask about on intake and I’ll also broach it again later on, because sometimes they’re not comfortable sharing that on intake.” (Doula)

“I can’t think if I’ve ever been asked that question in all my years of being a patient.” (NP)

“I definitely don’t remember my doctor that I see regularly asked me anything about.” (FG3)

***Context-dependent healthcare.*** Interviewees discussed how patient-professional dialogue on sexual assault appeared to be reliant on the healthcare setting. These shared experiences by healthcare professionals validate the discussion over the absence of screening for sexual assault within an obstetrical and gynecological healthcare setting. Professionals have highlighted the absence of sexual assault screening within obstetrical and gynecological healthcare appointments and illustrated its need as these professionals employed their form of screening depending on the patient they were seeing.

“In the ER, and I’m pretty sure this is nationwide, and at Planned Parenthood, I did too. We asked everybody ‘is there anybody hurting you or making you do things that you don’t want to do in your life right now?’” (NP)

“So, the experience I, that I’ve had like in an OB/GYN office, it actually was not something that was asked very often, even if the patient was alone. Which I thought was interesting. Because in the emergency department, you know, we ask every person, men, female. We ask everybody.” (NP)

“I took that from my emergency room training to that clinic. But it’s not part of, it’s not necessarily part of like the physicians [standard of care] or anything like that, it’s not built in that way.” (NP)

“Those are the ones that really red flags for me that she might be abused or sex trafficking or there might be something deeper going on that, you know.” (Nurse Manager)

### Traumatic healthcare: “The way that a clinical care provider treats a woman in labor can have a huge impact on her life forever. And she doesn’t understand that.” (Doula)

When discussing how healthcare can become traumatic for some patients, factors impacting this outcome presented themselves in two forms: (1) how healthcare professionals may prevent distressing care and (2) how professionals may be contributing to traumatic experiences in healthcare.

***Preventing distressing care.*** Many healthcare professionals shared how they utilize precautionary approaches when interacting with patients and probing on previous experiences. Interviewees outlined how healthcare professionals may approach sexual assault history by situating probing questions within conversations on how to best care for the patient while also showing respect for any sensitivities the patient may have. Focus group participants discussed cautionary approaches to care, which highlighted similar attitudes previously expressed by professionals.

“I would just say you mentioned you know, in your history… like we do have… counseling here if that would be helpful, or is there anything else… you want me to know so that I can best care for you that I can put in the chart?” (CNM)

“Sometimes it is, are there any triggers you have that I should worry about? Because then it’s kind of broad, that doesn’t necessarily mean sexual triggers. It just means, you know, overall life triggers. Or are there any experiences that you have that you think might make any part of labor and delivery a little bit more challenging for you, kind of again, with those open-ended questions, not necessarily directing it at sexual assault.” (Doula)

“We’ll write it on the birth plan… and we’ll say we don’t want to talk about this at the bottom. Here’s this disclaimer… these are their triggers don’t say some things… don’t say a certain phrase.” (Doula)

“I try to be really vocal through like all of that component for sure.” (CNM)

“really letting the woman kind of guide that [the physical exam]… and what that looks like and letting them just tell me what’s okay and what’s not okay, versus me saying, I’m going to check your cervix now.” (CNM)

“I make sure that every woman knows that you have to give consent for everything. If somebody’s saying I’m going to check your cervix. Now you can say No, thank you. And it doesn’t matter if they get shi**y about it. If they get hostile with you, you ask for a new nurse until you find one that is willing to respect your wishes.” (Doula)

“Being clear that they know that they can always tell someone to stop any sort of exam… pointing out parts of the process that they can ask to lead instead of having like a provider… physically do like for pap smear, it’s a really good example of that, like, patients don’t maybe always know that they can insert their own speculum if they want, like that sort of thing.” (CNM)

“Maybe just… having the exam itself could trigger stress for them… just always re-stating exactly what they’re about to do, what the patient should feel, what the patient will experience and just being very, very thorough.” (FG2)

***Healthcare professional-induced trauma.*** As healthcare professionals discussed precautionary care (i.e., explaining what they are doing and why during an examination), they also discussed patient outcomes when sensitivities are not considered. Interviewees also emphasized the impact professionals’ insensitivities can have on a woman during birthing, noting how previous sexual assault history impacts the process.

“I think it’s to find out if they’ve…ever been assaulted, to know their comfort level. Because, you know… you don’t want them to feel like they’re being re-assaulted.” (NP)

“I mean, that caused a lot of women to suffer post-traumatic stress from their birth… it isn’t even about that she had a C section it is about she was violated.” (NM, NP)

“She went through the C section, entire section with headphones and her hands over her eyes. She refused to look at her babies, she wouldn’t look at them in recovery. And that’s not because she’s a bad mom. It’s because she had horrible PTSD from a previous birth experience. And not understanding things like that can be devastating to these moms and it can have an impact on their ability to care for their infants.” (Doula)

“Those women tend to want a more hands-off pushing stage, because if there’s a bunch of hands inside them, touching them, rubbing them stretching them, it can cause rape flashbacks or sexual trauma flashbacks” (Doula)

“The way that a clinical care provider treats a woman in labor can have a huge impact on her life forever. And she doesn’t understand that.” (Doula)

“I mean yeah because you can throw somebody back into traumatic moments by doing things like if they don’t know.” (FG1)

In addition to discussing healthcare professional-induced trauma, interviewees spoke of patient consent and their distrust toward professionals for forcing services. Some participants highlighted how their colleagues ignore patient choice. Healthcare professionals stressed the significance of patient consent and choice when interacting with any patient whether they have a history of sexual assault or not, as these deliberate acts could inflict harm and create a culture of distrust. These narratives highlight the importance of interactions with patients, patient choice, and the way healthcare professionals approach screening and services.

“I don’t think these physicians realize what they’re doing to these women and that they are abusing them or, you know, going and doing a vaginal exam, all these unnecessary vaginal exams on women that the women think that they have to submit to.” (Doula)

“I have a lot of clients who will deny cervical exams to check for progression of labor, one because it really doesn’t get that much useful information. The cervix isn’t a crystal ball, you can go from zero to 10 in five minutes, or you could go from nine to 10 in six hours… Sometimes it can, you know, in certain situations, but as a rule of thumb, checking somebody dilation every two hours, isn’t really that beneficial or evidence-based… for those clients, they because the feeling of somebody else going inside their vagina can be very triggering to them.” (Doula)

“They tend to deny cervical exams. And then there’s this hostility with the providers, if they’re not understanding well, ‘I want this information,’ and the client then not feeling comfortable enough to say, ‘well, this is why I don’t want this.’” (Doula)

“that question [about sexual assault history] caught me off guard and then I had like a very traumatizing experience too, because the doctor was like aggressive with it. And, you know, I feel like how the question was asked would have definitely changed that experience.” (FG6)

### Impact on overall care. “Some of them won’t even go seek healthcare.” (NP)

Healthcare professionals described the impact a patient’s history of trauma has when providing care, and the long-term implications that stem from it. Focus group participants continued to describe the discomfort that can arise in healthcare after previous traumatic experiences.

**Influence of trauma on health.** As professionals discussed the impact sexual assault may have on a patient’s healthcare, it became apparent that unaddressed trauma appeared to impact overall health. Many interviewees discussed patient discomfort and hesitancy in seeking care while also citing the limits trauma places on care. Healthcare professionals also described the amplified discomfort patients with previous sexual assault experience may feel due to routine reproductive health examinations. These professionals clarified the impact sexually violent experiences have on a patient’s health by citing hesitancy seeking care, and the fear connected to examinations. Healthcare professionals further emphasized this point noting the physical and mental health implications of sexual assault trauma.

“It prevents them from coming in or, you know, kind of limits them on contraception options… they’ll usually tell us, some people will talk about it. Some people will just say, ‘you know, I’ve had trauma in my past and I do not want to come into the office.’” (Clinical Pharm Specialist)

“yeah panic attacks. Either like feeling uncomfortable with exams, who’s in the room you know who touches them, cares for them?” (CNM)

“let’s say the NuvaRing most women with trauma regarding like their vagina don’t really want to remove and insert something you know, like, regularly from their vagina, vulva, so I think it definitely can affect like how quick they are to seek care… If they feel like they’re going to be required to, you know, have a vaginal exam or something breast exam, that kind of stuff, too. I’m sure, it’ll be traumatic as well.” (CNM)

One interviewee stated, “they would come in a lot with you know, decreased libido problems or they would be concerned that their thyroid wasn’t working right or whatever, because they just had this aversion to having sex and they just didn’t like sex because they had had a bad experience.” (NP)

Healthcare professionals continued to list the impacts sexual assault experiences have on healthcare; some citing issues related to pregnancy. Professionals’ experiences with patients highlight the impact traumatic experiences have on a patient’s health, the services they seek, and how care may be delivered. Focus group discussions also revealed how sexual assault can impact patient decision-making and overall health.

“they may not want to breastfeed or so or like having cervical exams may be very uncomfortable for them” (Doula)

“Some of it is a power thing to a lot of women that I’ve seen that are domestic abuse survivors are more apt to want to have unmedicated births because they feel like it’s a challenge. It’s an empowering thing. They can say they did something really hard. And it kind of makes them feel better about themselves makes them feel stronger.” (Doula)

“I know people who, like felt like they needed to be on some sort of hormonal birth control. Like they were sexually assaulted, and the person didn’t use a condom or like, if they had a history of that happening and they didn’t want that to happen again. They made sure they were on something that they could rely on.” (FG6)

***Professional Interactions.*** Healthcare professionals emphasized the importance of knowing about a patient’s history of sexual assault so they may provide better care. These conversations with professionals described how and what communication issues may arise when these situations go unaddressed. Focus group participants discussed their thoughts on the importance of healthcare professionals in listening and communicating.

“these are things that can kind of clue you into, okay, there’s something else here. It is surprising how many women in this context, once you build a relationship with them, will tell you.” (Doula)

“I think there’s a lack of communication as far as you know, or education, I should say, as far as okay, once you ask that question, they say ‘yes’ and then what, you know? what, what should we say? Or how should we say it? Or there’s really no education for providers as what we’re supposed to do, you know, once they say ‘yes’ to that.” (NP)

“we definitely can be a supportive person to talk to, we always suggest we would refer out to a therapist if they weren’t already utilizing one. But we have resources, you know, at our disposal to get them to the right person.” (Doula)

“I just kind of asked her, like, I would like to put some information in the chart so that people are sensitive to what has happened to you in the past. So, if there’s any, you know, and I don’t have to put I can put in there, whatever you want people to know, or don’t want them to know, however you want me to put it in there just so that they like that you don’t want lots of actual exams that you don’t want to male provider, those types of things.” (CNM)

“You see fight or flight for those people in situations, but to a clinician are everyday things so they don’t understand what’s happening… of course that it escalates because the clinician thinks they’re being ignorant or negligent or non-compliant or you know, and no, you have a terrified person.” (NM, NP)

“you know what we can get from this is being really good at communicating with your patient. I think that’s more vital than anything and just like trying to be really empathetic and focusing on like, making them feel comfortable but also in a way that’s encouraging them.” (FG2)

***Male Healthcare Professionals.*** Interview and focus group participants frequently addressed male professionals’ understanding of sexual trauma and how it presents itself in healthcare, including their lack of capacity to address trauma when presented with it. While male professionals’ understanding and attention toward sexual assault was often discussed, the comfortability that female survivors have with male professionals presented itself just as frequently.

“One male provider I have talked to before and I used the term traumatic birth and he got really upset. He said, ‘I’ll show you trauma. If the baby dies, it’s trauma. If the mom has to have a hysterectomy, it’s trauma’ and he got really upset and I’m like, ‘No, I understand those things are trauma but what I’m telling you is there’s traumas that you can’t see, that you can’t quantify. And you’re not caring about those traumas, and those have an impact on life.’” (Doula)

“I think honestly, he might have just thought it was like his eyes on the patient were enough of a screening.” (NP)

“I recently had someone come to me for a pap smear, who had been seen at like a local community health center, mostly and kind of like jumped around a lot and her primary care, it was male, and his note said something like ‘she prefers a female to do her pap smear’ and I like looked through history, and she had had an elective abortion at age 13. And so, like I started off the office visit, just, you know, saying it like, it’s okay if, she was like in her fifties, like, it’s okay, if you don’t want to talk about this, but I just wanted to know, since part of this exam will be a pelvic exam if you’ve ever been sexually abused, and if that’s something that I need to know about to be able to complete your exam.” (CNM)

“She just admitted to me that she had been raped in the past. And she was asking if there were any male providers that I was, you know, just educating there, there were not.” (CNM)

“I’m sure that play like a huge part in like, whether they choose a female or male doctor. You know, if it’s a woman who’s been raped by a man like she’s probably not going to want a male doctor, right and vice versa. Yeah. Huge role in future healthcare decisions.” (FG2)

“A man wouldn’t think to ask that cause he’s not a woman.” (FG3)

Overall, there was an overlapping perception among this sample of both professional and non-professional participants that male healthcare professionals struggle not only to provide a safe environment for survivors to share their experiences, but also to understand traumatic experiences and adequately address them.

## Discussion

Professionals and patients highlighted the absence of sexual assault screening within obstetrical and gynecological healthcare appointments and illustrated its need, including various ways professionals use their screening practices and tools. Participants also noted how professionals can use strategies during routine visits to prevent traumatic experiences. However, instances were also noted where professionals contribute to trauma through the care they provide. These experiences may create distrust among patients and professionals, thus impacting current and future care. Lastly, interview and focus group participants noted how a history of sexual assault could impact patient-professional communication and rapport, including how a professional’s demographics influence the environment and care quality.

Findings revealed healthcare professionals’ and non-healthcare participants’ perceptions and experiences of the impact of sexual assault, including undisclosed sexual assault, on obstetrical and gynecological healthcare. These impacts spanned negative patient reactions during gynecological and obstetrical care (e.g., panic attacks, discomfort), reduced follow-up, and impacts on healthcare choices (e.g., choosing not to breastfeed, preferring non-medicated childbirth, contraceptive choice). Prior research has identified reduced healthcare-seeking, follow-up, and suboptimal clinical experiences resulting from sexual assault history [3], supporting this sample’s findings. Though extant literature identified the influence of prior trauma (emotional, physical, or sexual abuse) on contraceptive method choice and continuation [3, 15], healthcare professionals also described how sexual assault affected other healthcare decisions. This finding highlights the importance of screening for sexual assault by engaging patients in provider-initiated conversations to discuss patients’ sexual assault history and outline potential needs before, during, and after a healthcare appointment, while ensuring comfort and consent.

Building trauma-informed care into obstetrical and gynecological healthcare that is intuitive to patient experiences, needs, and priorities may improve women’s care, health outcomes, and empower women in their gynecological and birth choices [46, 47]. Doing so requires equipping healthcare professionals with the language and tools needed to regularly engage women in sexual assault screening in a patient-centered way. By developing and strengthening relationships, healthcare professionals were better able to identify possible sexual assault among their patients, broach conversations about it, and make women feel more comfortable sharing. This finding aligns with prior research on the importance of patient relationship on disclosure [46, 47]. Providing possible screening verbiage and education for healthcare professionals regarding what to do after a disclosure is made and how to build this context into a patient’s care continuum is critical for enhancing obstetrical and gynecological healthcare experiences [57]. Prior work has identified strategies for developing trauma-informed care practice [60]. For example, some women who experienced sexual assault by a man may desire a woman healthcare professional to avoid re-traumatization. This builds on prior research suggesting women select their providers to prepare for, navigate, and recover from healthcare experiences if they have experienced prior trauma [46, 47]. This is a contextual factor specific to each patient with a history of sexual assault but should be considered when identifying obstetrical and gynecological healthcare needs at the onset of an appointment to ensure a more positive care experience in the long term.

One strength of this study was the inclusion of doulas. Doulas may gain additional patient context that a healthcare professional can use, with patient permission, to increase sensitivity to women’s needs in their birth experience. This aligns with prior work that found women may bring support people to appointments to help them navigate potentially difficult healthcare experiences [46, 47]. Thus, opportunities exist to bridge the gap between routine trauma-informed care and current healthcare practice and build provider-driven trauma-informed care capacity by including other types of healthcare professionals in women’s care teams. These healthcare professionals (e.g., doulas, clinical pharmacists) can also build these relationships with women and advocate, support, empower, and/or share information─ with patient consent─ during qualifying healthcare appointments to establish consistent, quality care.

### Limitations and future research

Results should be interpreted in the context of some limitations. Results may not be generalizable across different populations, including other geographic locations or sociodemographic groups, though these findings may transfer to other contexts and samples. Because of the social nature of focus groups, some participants may not have shared their experiences due to desirability bias. Complete confidentiality could not be guaranteed due to the social environment, and the sample was a convenience sample; healthcare professionals also self-reported their behaviors, which may have led to social desirability and recall bias. Additionally, most professionals were white and female, which aligns with healthcare professional demographics in Indiana, but limits generalizability to other locations. There may have been selection bias and differences in interview experiences due some being conducting in-person, while others were conducted via phone. Future work should consider the strengths and limitations of in-person vs., virtual or phone interviews [79].

Despite these limitations, this study had many strengths. Qualitative methodology allowed for robust insight into rich experiences. Different healthcare professional-type and community healthcare settings allowed for triangulation of ideas and consistent themes across professional experiences. Varied recruitment strategies also assisted in enrolling healthcare professionals from different women’s reproductive healthcare sub-fields. Research experts and healthcare professionals reviewed interview and focus group guides. Future research should further identify what impacts healthcare professionals’ decisions to discuss and screen for a history of sexual assault during obstetrical and gynecological healthcare appointments, beyond those identified in this study, and examine their role in sexual assault prevention and recovery. Additionally, future research should explore the acceptability of the sexual assault screening procedures suggested by participants in enhancing patient involvement in their obstetrical and gynecological healthcare appointments. Finally, future research should further explore experiences with and preferences for providers based on the provider’s personal characteristics, such as gender.

### Implications

Community healthcare professionals are critical in discussing sexual assault during obstetrical and gynecological healthcare appointments. Even when communication barriers exist, demonstrating listening offers professionals one way to ensure patients feel valued, safe, and secure. Inquiring into sexual assault history and consenting prior to engaging with patients may allow professionals to personalize care and support patients who may be traumatized. Healthcare professionals should keep in mind patients may have prior negative healthcare interactions that impact their current views and behaviors. Thus, professionals should facilitate positive engagement via communication strategies, such as building quick rapport, refraining from abruptly ending conversations, validating experiences, and engaging in demonstrative listening. Community healthcare professionals may be one touchpoint for connecting survivors to care and wraparound services.

## Conclusions

Findings offered insight into actionable and practical strategies for enhancing sexual assault screening and discussions in community-based obstetrical and gynecological healthcare appointments. Incorporating healthcare professionals’ and patients experiences and preferences for sexual assault-related discussions during routine obstetrical and gynecological care can assist in sexual assault prevention and treatment efforts, improve patient-professional rapport, and yield better health outcomes.

## Data Availability

Data and interview guide available by request. Please email Andrea L. DeMaria at ademaria@purdue.edu.
